# Prognostic value of postoperative glucose-to-lymphocyte ratio and visceral fat index in non-diabetic gastric cancer patients: a mediation and modeling study

**DOI:** 10.3389/fnut.2026.1838308

**Published:** 2026-10-23

**Authors:** Min Lai, Ning Lan, Yujie Zhou, Wenzhen Yuan

**Affiliations:** 1 The First School of Clinical Medicine, Lanzhou University, Lanzhou, China; 2 Department of Radiation Oncology, Gansu Provincial Maternity and Child Health Hospital, Lanzhou, China; 3 Department of Oncology, Gansu Provincial Maternity and Child Health Hospital, Lanzhou, China; 4 Department of Surgical Oncology, The First Hospital of Lanzhou University, Lanzhou, China

**Keywords:** glucose-lymphocyte ratio, mediation effect, nomogram, overall survival, visceral adipose tissue index

## Abstract

**Background:**

Prognostic assessment in gastric cancer (GC) remains challenging, as TNM staging has limited utility for individualized risk stratification. Systemic inflammation, metabolic status, immune function, and body composition are known to influence survival outcomes. This study aimed to evaluate the prognostic value of preoperative visceral adipose tissue index (VATI) and postoperative day 1 (POD1) glucose-to-lymphocyte ratio (GLR) in non-diabetic GC patients, and to develop an integrated prognostic model.

**Methods:**

This retrospective cohort study analyzed data from 339 GC patients, who were randomly assigned to a training cohort (*n* = 272) and an internal validation cohort (*n* = 67) in an 8:2 ratio using a computer-generated random sampling method. Cox regression was performed to identify independent predictors of overall survival (OS). Causal mediation analysis using a linear mediator model and a parametric accelerated failure-time survival model was performed to assess whether POD1 GLR mediated the association between preoperative VATI and OS. A TGV nomogram, integrating TNM stage, neoadjuvant chemotherapy (NAC), GLR, and VATI, was developed and validated using the concordance index (C-index), receiver operating characteristic (ROC) curves, calibration plots, and decision curve analysis (DCA).

**Results:**

Multivariable Cox regression analysis showed that NAC, advanced pTNM stage, high POD1 GLR, and low VATI were independently associated with poorer OS (*p* < 0.05). Mediation analysis showed no significant indirect effect of POD1 GLR on the VATI–OS relationship (ACME, −3.82 days; 95% CI, −189.75 to 105.37; *p* = 0.899). Similar findings were obtained in the POD1 landmark and surgically adjusted sensitivity analyses. The TGV nomogram achieved 3- and 5-year AUCs of 0.850 and 0.798 in the training cohort and 0.785 and 0.729 in the internal validation cohort, respectively. The corresponding AUCs for TNM staging alone were 0.769 and 0.702 in the training cohort and 0.782 and 0.642 in the internal validation cohort. Training-cohort DCA suggested a higher net benefit for the TGV model than TNM staging across much of the evaluated threshold range at both 3 and 5 years.

**Conclusion:**

In this retrospective cohort of non-diabetic GC patients, low preoperative VATI and high POD1 GLR were independently associated with poorer OS. Mediation analysis provided no statistically significant evidence that POD1 GLR mediated the association between VATI and OS. The integrated model combining pTNM stage, NAC, GLR, and VATI showed potential for risk stratification, although its incremental prognostic value over established TNM-based models requires confirmation in independent external cohorts.

## Introduction

Gastric cancer (GC) is the fifth most common malignancy and the third leading cause of cancer-related mortality globally (1). Despite significant advances in diagnostic and therapeutic strategies, the prognosis for GC patients remains suboptimal (2). The TNM staging system is the standard tool for prognostic assessment; however, it primarily relies on anatomical and histopathological characteristics, without adequately accounting for the biological factors influencing patient outcomes (3). This limitation contributes to significant prognostic variability among patients with the same disease stage. Thus, there is a pressing need for a comprehensive prognostic model that integrates multiple dimensions of biological data to enhance the accuracy and clinical utility of GC prognosis.

Immune function, inflammation, metabolism, and body composition are well-established determinants of survival in cancer patients (4). While numerous studies have employed inflammatory, immune, and metabolic markers for prognostic purposes, these markers are often unidimensional and fail to capture the full complexity of the body’s biological condition (5–9). Postoperative hyperglycemia, a frequent metabolic response following radical gastrectomy, is commonly observed in non-diabetic patients and is associated with an increased incidence of complications and poorer survival outcomes (10, 11). The underlying mechanisms are believed to involve stress hormone-induced pro-inflammatory responses and insulin resistance (12). Interestingly, non-diabetic patients with comparable levels of postoperative hyperglycemia exhibit worse outcomes than diabetic patients, suggesting a distinct impact of hyperglycemia in non-diabetic individuals (10, 13, 14). In this context, the glucose-to-lymphocyte ratio (GLR), which integrates both metabolic and immunological markers, has shown prognostic value in various solid tumors (13). However, evidence supporting its application in non-diabetic GC patients is limited, and its potential interaction with body composition indices remains underexplored (15).

Body composition parameters, such as adipose tissue and skeletal muscle, provide critical insights into a patient’s nutritional and metabolic status, as well as their physiological reserves (16). Preoperative abdominal computed tomography (CT) enables the quantification of the visceral adipose tissue index (VATI) and skeletal muscle index, offering a reliable measure of a patient’s nutritional compensatory capacity (10, 17). Reduced fat reserves have been consistently linked to poor postoperative outcomes in GC patients (16, 17). Previous studies have suggested that postoperative hyperglycemia may partially mediate the relationship between VATI and overall survival (OS) in non-diabetic GC patients (18). However, these studies have been limited to single glycemic markers and have not incorporated immune status. Given that surgical stress induces both metabolic and immune dysfunction, the role of GLR—reflecting both glucose and lymphocyte dynamics—remains unclear in mediating the impact of VATI on survival.

This study aims to assess the prognostic impact of preoperative body composition and postoperative GLR on OS in non-diabetic GC patients. We further seek to examine the interrelationship between these factors through mediation analysis. Finally, we aim to develop an integrated nomogram that incorporates inflammatory, metabolic, immune, and nutritional factors to improve prognostic accuracy and clinical utility for GC patients.

## Methods

### Patients

This retrospective cohort study analyzed clinical data from patients who underwent radical gastrectomy for GC at the First Hospital of Lanzhou University between Mar 2017 and June 2021. The present study and our previously published BMC Cancer study were derived from the same initial cohort of 445 patients (18). Following the application of study-specific eligibility criteria, the final analytical cohorts comprised 339 and 349 patients, respectively, including 325 patients common to both studies.

The inclusion criteria were: (1) Age 18–80 years; (2) Histologically confirmed GC; (3) Underwent D2 lymph node dissection; (4) Normal liver and kidney function; (5) Stage I–III based on the 8th edition of the AJCC classification. Exclusion criteria were: (1) History of diabetes or admission random venous blood glucose >11.1 mmol/L (200 mg/dL); (2) Postoperative ICU transfer; (3) History of other malignancies; (4) Incomplete clinical or imaging data (Figure 1). HbA1c was not used as an exclusion criterion because it was not routinely measured at admission in most patients without a known history of diabetes. For prognostic model development and internal validation, the 339 eligible patients were randomly assigned to the training cohort or validation cohort using SPSS 22.0’s random number generation function. A random seed of 123 was set, and the ‘Select Cases’ function with the ‘Random Sample’ option was used to allocate 20% of cases to the validation cohort, with the remaining 80% automatically assigned to the training cohort. This training–validation split was used only for prognostic model development and internal validation; association, survival, and mediation analyses were performed using the complete eligible cohort of 339 patients.

**Figure 1:**
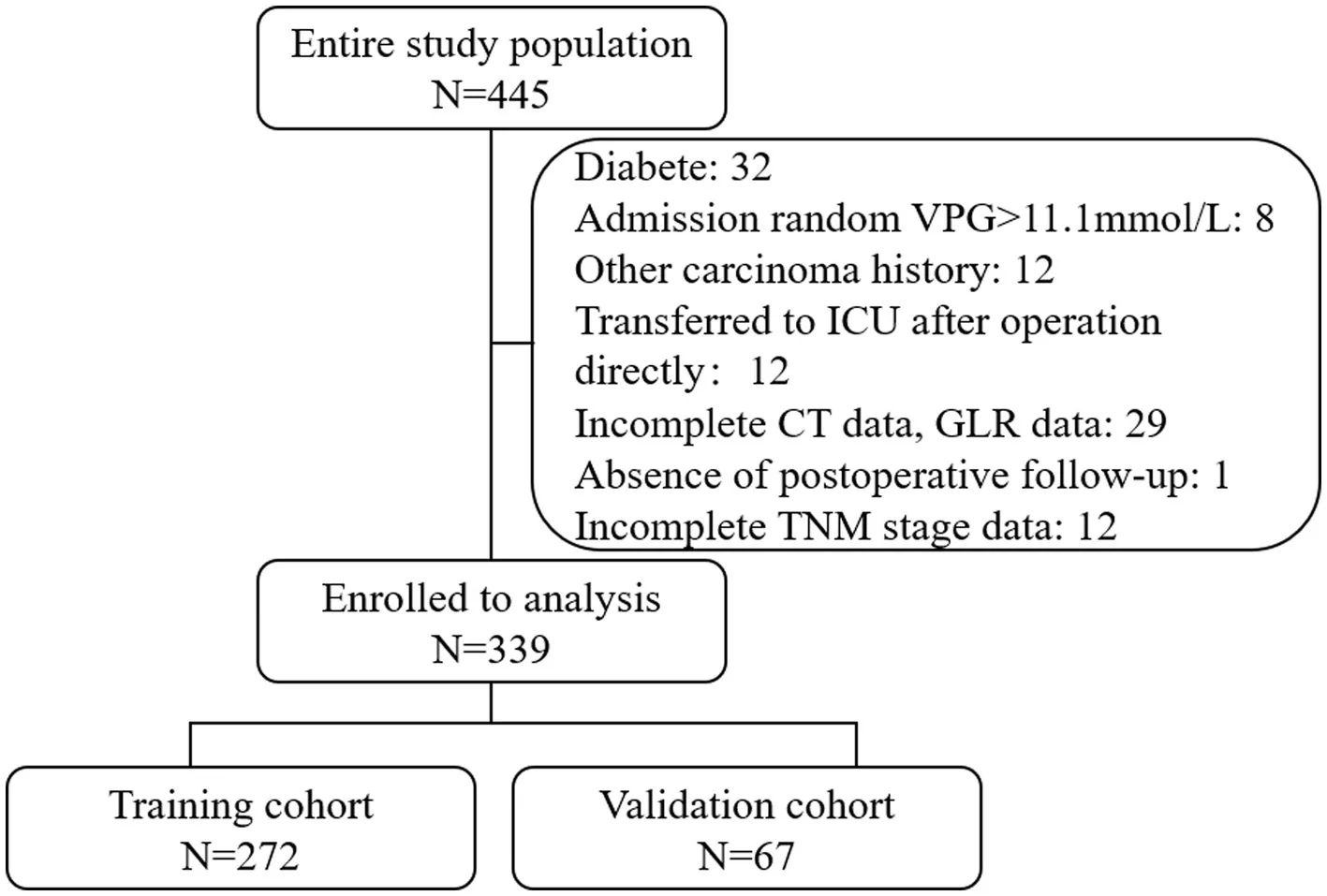
Flow diagram.

### Data collection

Clinicopathological and biochemical data were retrospectively extracted from the Hospital Information System (HIS). These parameters reflect the standard preoperative assessment and postoperative monitoring protocol routinely implemented at our institution to evaluate surgical indications and patient recovery. The data collected from patients included: (1) General characteristics: age, sex, height, weight, body mass index (BMI), neoadjuvant chemotherapy status, pTNM staging, histological grade, fasting venous blood glucose and peripheral blood lymphocyte count measured on postoperative day 1 (POD1), approximately 24 h after surgery; (2) Surgical-related data: surgical approach, gastrointestinal reconstruction method, and extent of gastrectomy. GLR was calculated as fasting venous blood glucose (mmol/L) divided by lymphocyte count (×10^9^/L) (15). The Prognostic Nutritional Index (PNI) was calculated as albumin (g/L) + 5 × total lymphocyte count (×10^9^/L). Hypoproteinemia was defined as albumin <35 g/L.

### Body composition assessment

Preoperative contrast-enhanced abdominal CT imaging, performed within 1 month prior to surgery, is mandatory for tumor localization and TNM staging. For this study, archived DICOM images were retrieved from the hospital’s Information Center using unique patient identifiers and CT accession numbers. Contrast-enhanced phase images at the L3 level were selected, and body composition was analyzed using Slice-O-Matic 5.0 software. The CT value (HU) ranges for visceral fat, subcutaneous fat, and skeletal muscle were −150 to −50 HU, −190 to −30 HU, and −29 to +150 HU, respectively. The software automatically calculated the areas of each region, including skeletal muscle area, visceral fat area, and subcutaneous fat area. These areas were then height-standardized to derive the VATI (cm^2^/m^2^), subcutaneous adipose tissue index (SATI; cm^2^/m^2^), and skeletal muscle index (SMI; cm^2^/m^2^). Skeletal muscle density (SMD) was determined as the average CT value of skeletal muscle, automatically calculated by the software. Sarcopenia was diagnosed based on the following criteria: SMI < 40.8 cm^2^/m^2^ for men and <34.9 cm^2^/m^2^ for women (19). Skeletal muscle fat infiltration was diagnosed with the following thresholds: SMD < 38.5 HU for men and <28.6 HU for women (20). All CT images were independently analyzed by the same radiologist.

OS was defined as the time from the date of surgery to the date of the last follow-up or death. Patients who remained alive at the last follow-up or were lost to follow-up were censored at the date of their last confirmed contact. The clinical follow-up database was frozen on April 11, 2023.

### Causal mediation analysis

Causal mediation analysis was performed to assess whether POD1 GLR mediated the association between preoperative VATI and OS. VATI and POD1 GLR were entered as continuous variables and standardized to z-scores. The mediator model used linear regression, whereas the survival outcome model used a parametric accelerated failure-time (AFT) model. Both models were adjusted for age, sex, neoadjuvant chemotherapy, pathological TNM stage, admission venous blood glucose, and preoperative neutrophil-to-lymphocyte ratio. Candidate AFT distributions were compared using AIC and BIC, and the log-logistic model was selected for the primary analysis.

Mediation effects were estimated using mediation::mediate(), with lm() for the mediator model and survival::survreg() for the outcome model. Average causal mediation effects (ACME), average direct effects (ADE), total effects, and proportions mediated were estimated using 5,000 nonparametric bootstrap replications with percentile 95% confidence intervals. Effects were expressed on the model-predicted survival-time scale rather than as hazard ratios.

Because GLR was measured on POD1 whereas OS was defined from the date of surgery, a POD1 landmark sensitivity analysis was performed. A further sensitivity analysis additionally adjusted for surgical approach and extent of gastrectomy. Causal interpretation assumes correct model specification, positivity, and no unmeasured confounding.

### Prognostic model development and performance assessment

Prognostic factors identified in the overall-cohort multivariable Cox analysis were used to construct the prognostic model in the training cohort. Reference models based on TNM staging alone or combined with PNI, NLR, or PLR were also fitted in the training cohort. All training-derived models were subsequently applied to the internal validation cohort without refitting.

Model discrimination was assessed using Harrell’s C-index and 3- and 5-year cumulative/dynamic time-dependent receiver operating characteristic (ROC) analysis with inverse probability of censoring weighting using the time ROC package. Calibration was assessed by grouping patients into tertiles according to model-predicted survival probabilities in both the training and internal validation cohorts; within each tertile, Kaplan–Meier-estimated observed survival was compared with the mean model-predicted survival probability at 3 and 5 years.

### Statistical analysis

The optimal cutoff value was determined using X-tile 3.6.1 software (21). Continuous variables following a normal distribution are expressed as mean ± standard deviation (x ± s), and comparisons between groups were performed using the independent samples t-test. For non-normally distributed data, values are expressed as median and interquartile range [M(IQR)], with comparisons made using the Kruskal-Wallis test. Categorical variables are presented as frequencies (percentages), and comparisons between groups were conducted using the chi-square test or Fisher’s exact test. Survival analysis was performed using the Kaplan–Meier method, and group comparisons were made using the log-rank test. Univariable Cox proportional hazards regression was used to assess factors associated with OS. Variables with *p* < 0.10 in the univariable analysis were entered into the multivariable Cox regression model, and hazard ratios (HRs) with 95% confidence intervals (CIs) were reported.

All statistical analyses were conducted using SPSS 22.0 and R 4.5.3 software, with a significance threshold set at *p* < 0.05.

## Results

### Patient characteristics

There were no significant differences in demographic and clinical-pathological characteristics between the training group (*n* = 272) and the validation group (*n* = 67) (*p* > 0.05) (Table 1). These characteristics included age (≥65 years), gender (male), neoadjuvant chemotherapy (NAC), nutritional indices (PNI, GLR, SMI, SMD, VATI, SATI), TNM staging, histological grade, surgical approach, gastrointestinal reconstruction method, and extent of gastrectomy. There were no significant differences in admission venous blood glucose (*p* = 0.340) or POD1 blood glucose (*p* = 0.666) between the training and validation groups. The NAC group had a higher proportion of open surgeries (75.9% vs. 60.1%, *p* = 0.004) and total gastrectomies (65.5% vs. 35.4%, *p* < 0.001) (Supplementary Table 1).

**Table 1:** Demographic and clinicopathological characteristics.

Variable	Training cohort	Validation cohort	*p*
	*N* = 272	*N* = 67	
Age			0.587
<65 years	188 (69.1)	44 (65.7)	
≥65 [*n* (%)]	84 (30.9)	23 (34.3)	
Male [*n* (%)]	210 (77.2)	53 (79.1)	0.739
BMI [*n* (%)]			0.624
<18.5	31 (11.4)	7 (10.4)	
18.5–24	170 (62.5)	46 (68.7)	
≥24	71 (26.1)	14 (20.9)	
NAC [*n* (%)]			0.580
No	177 (65.1)	46 (68.7)	
Yes	95 (34.9)	21 (31.3)	
Chemotherapy Regimen [*n* (%)]			0.715
SOX	80 (84.2)	17 (81.0)	
XELOX	3 (3.2)	0 (0)	
FOLFOX	1 (1.1)	0 (0)	
Other	11 (11.5)	4 (19.0)	
Albumin, g/L [M (IQR)]	43.10 (5.00)	43.5 (4.70)	0.464
Hypoproteinemia [*n* (%)]	71 (26.1)	20 (29.9)	0.535
HDL[M(IQR)]	1.07 (0.42)	1.09 (0.40)	0.420
Admission venous blood glucose (mmol/L) [M(IQR)]	5.01 (0.98)	5.26 (0.90)	0.340
POD1 blood glucose (mmol/L) [M(IQR)]	6.3 (1.60)	6.4 (1.35)	0.666
PNI [*n* (%)]			0.633
L- PNI	44 (16.2)	11 (16.4)	
M- PNI	146 (53.7)	32 (47.8)	
H- PNI	82 (30.1)	24 (35.8)	
GLR [*n* (%)]			0.911
L- GLR	67 (24.6)	18 (26.9)	
M- GLR	174 (64.0)	41 (61.2)	
H- GLR	31 (11.4)	8 (11.9)	
SMI (x ± s)	45.66 ± 8.29	45.49 ± 6.68	0.885
Sarcopenia [*n* (%)]	106 (39.0)	27 (40.3)	0.842
SMD [M (IQR)]	43.41 (7.62)	43.16 (7.76)	0.420
VATI, cm^2^/m^2^ [*n* (%)]			0.224
L- VATI	60 (22.1)	9 (13.4)	
M- VATI	95 (34.9)	23 (34.3)	
H- VATI	117 (43.0)	35 (52.2)	
SATI [*n* (%)]			0.463
L- SATI	40 (14.7)	6 (9.0)	
M- SATI	165 (60.7)	44 (65.7)	
H- SATI	67 (24.6)	17 (25.4)	
pTNM [*n* (%)]			0.212
I	77 (28.3)	26 (38.8)	
II	76 (27.9)	14 (20.9)	
III	119 (43.8)	27 (40.3)	
Histological grade [*n* (%)]			0.552
High middle differentiation	11 (4.0)	1 (1.5)	
Middle differentiation	36 (13.2)	6 (9.0)	
Middle low differentiation	211 (77.6)	56 (83.6)	
Specific pathological type	14 (5.1)	4 (6.0)	
Operation approach [*n* (%)]			0.409
Open	181 (66.5)	41 (61.2)	
Laparoscopic	91(33.5)	26 (38.8)	
Reconstruction [*n* (%)]			0.124
Billroth I	12 (4.4)	6 (9.0)	
Billroth II	55(20.2)	8 (11.9)	
Roux-en-Y	205(75.4)	53 (79.1)	
Gastrectomy scope [*n* (%)]			0.955
Proximal	3 (1.1)	1 (1.5)	
Distal	144(52.9)	36 (53.7)	
Total	125(46.0)	30 (44.8)	

BMI, Body mass index; NAC, Neoadjuvant chemotherapy; HDL, High-density lipoprotein; PNI, Prognostic nutritional index; GLR, Glucose-to-lymphocyte ratio; SMI, Skeletal muscle index; SMD, Skeletal muscle density; VATI, Visceral adipose tissue index; SATI, Subcutaneous adipose tissue index; TNM stage, Tumor-node-metastasis stage.

To assess the impact of the COVID-19 pandemic, a comparative analysis was conducted between the pre-pandemic (March 2017 to February 2020; *n* = 181) and pandemic (March 2020 to June 2021; *n* = 158) periods. No significant differences were observed in BMI (*p* = 0.172), VATI (*p* = 0.081), PNI (*p* = 0.561), or pTNM stage distribution (*p* = 0.089) (Supplementary Table 2).

The optimal cutoff values for GLR, PNI, SATI, and VATI were determined in the training set (*n* = 272) using X-tile software (Supplementary Figure 1). GLR was categorized into three groups: low (≤5.56), intermediate (5.56 < GLR ≤ 14.38), and high (>14.38); VATI was categorized into three groups: low (≤5.21), intermediate (5.21 < VATI ≤21.79), and high (>21.79) (Table 1). Correlation analyses revealed significant positive correlations between VATI and nutritional/anthropometric parameters, including BMI, SMI, and albumin (*p* < 0.001) (Supplementary Figure 2). The mean SMI increased progressively across VATI categories, with the highest value observed in the high-VATI group (Supplementary Table 3). However, no significant associations were found between VATI and baseline systemic inflammatory markers, including NLR, LMR, and PLR (*p* > 0.05) (Supplementary Figure 2).

### Univariable and multivariable cox regression analyses

Univariable analysis revealed several factors significantly associated with OS, including age ≥65 years (HR = 1.782, 95% CI, 1.139–2.789; *p* = 0.011), NAC (HR = 2.452, 95% CI, 1.570–3.828; *p* < 0.001), the high GLR group (HR = 3.509, 95% CI, 1.641–7.502; *p* = 0.001), pTNM stage II (HR = 3.640, 95% CI, 1.537–8.621; *p* = 0.003), pTNM stage III (HR = 6.629, 95% CI, 3.000–14.650; *p* < 0.001), the high SATI group (HR = 0.287, 95% CI, 0.132–0.621; *p* = 0.002), and the low VATI group (HR = 2.401, 95% CI, 1.400–4.118; *p* = 0.001) (Table 2).

**Table 2:** Univariable and multivariable Cox regression analyses for overall survival in the entire cohort.

Variable	Univariate analysis		Multivariate analysis	
	HR (95%CI)	*p*	HR (95%CI)	*p*
Age				
<65 years	Reference		Reference	
≥65 years	**1.782 (1.139–2.789)**	**0.011**	1.430 (0.901–2.270)	0.129
BMI				
<18.5	Reference		Reference	
18.5–24	0.583 (0.315–1.078)	0.085	0.749 (0.391–1.435)	0.383
≥24	0.567 (0.278–1.158)	0.119	1.039 (0.452–2.386)	0.928
NAC				
No	Reference		Reference	
Yes	**2.452 (1.570–3.828)**	**<0.001**	**2.622 (1.641–4.191)**	**<0.001**
GLR				
L-GLR	Reference		Reference	
M-GLR	1.800 (0.959–3.377)	0.067	1.593 (0.835–3.037)	0.158
H-GLR	**3.509 (1.641–7.502)**	**0.001**	**2.569 (1.167–5.657)**	**0.019**
pTNM				
I	Reference		Reference	
II	**3.640 (1.537–8.621)**	**0.003**	**3.270 (1.358–7.876)**	**0.008**
III	**6.629 (3.000–14.650)**	**<0.001**	**6.329 (2.825–14.177)**	**<0.001**
Histological grade				
High middle differentiation	Reference			
Middle differentiation	0.758 (0.147–3.909)	0.741		
Middle low differentiation	2.162 (0.530–8.823)	0.282		
Specific pathological type	0.938 (0.132–6.663)	0.949		
SMI	0.979 (0.953–1.007)	0.136		
Sarcopenia	1.370 (0.877–2.138)	0.166		
SMD	0.985 (0.952–1.019)	0.379		
Myosteatosis	1.447 (0.899–2.329)	0.128		
SATI				
L-SATI	Reference		Reference	
M-SATI	0.621 (0.362–1.064)	0.083	0.951 (0.450–2.008)	0.895
H-SATI	**0.287 (0.132–0.621)**	**0.002**	0.578 (0.198–1.685)	0.315
VATI				
H-VATI	Reference		Reference	
M-VATI	1.308 (0.751–2.278)	0.342	1.088 (0.576–2.055)	0.796
L-VATI	**2.401 (1.400–4.118)**	**0.001**	**2.341 (1.058–5.178)**	**0.036**
Complication	0.751 (0.406–1.389)	0.361		

BMI, Body mass index; NAC, Neoadjuvant chemotherapy; GLR, Glucose-to-lymphocyte ratio; TNM stage, Tumor-node-metastasis stage, SMI, Skeletal muscle index; SMD, Skeletal muscle density; SATI, Subcutaneous adipose tissue index; VATI, Visceral adipose tissue index. Bold values indicate statistically significant hazard ratios and corresponding p-values (*p* < 0.05).

Variables with *p* < 0.10 in the univariable analysis were included in the multivariable analysis. The results showed that NAC (HR = 2.622, 95% CI, 1.641–4.191; *p* < 0.001), the high GLR group (HR = 2.569, 95% CI, 1.167–5.657; *p* = 0.019), pTNM stage II (HR = 3.270, 95% CI, 1.358–7.876; *p* = 0.008), pTNM stage III (HR = 6.329, 95% CI, 2.825–14.177; *p* < 0.001), and the low VATI group (HR = 2.341, 95% CI, 1.058–5.178; *p* = 0.036) remained independently associated with OS (Table 2).

### Kaplan–Meier survival analysis

The median follow-up duration for the entire cohort, estimated by the reverse Kaplan–Meier method, was 37.9 months (95% CI, 34.7–46.4 months). During the follow-up period, the median OS was not reached in the entire cohort. The 3-year OS rate was 74.3% and the 5-year OS rate was 66.9%.

Survival analysis revealed that both GLR and VATI were significantly associated with OS (*p* < 0.05). High GLR and low VATI were each associated with markedly poorer OS (Supplementary Figures 3A,B).

### Mediation effect model

Among the candidate parametric AFT distributions, the log-logistic model yielded the lowest AIC (1629.985), while its BIC was close to the minimum (1672.071, ΔBIC = 0.575); it was therefore selected for the primary mediation analysis (Supplementary Table 4). No statistically significant indirect effect of POD1 GLR was observed on the association between preoperative VATI and OS (ACME, −3.82 days; 95% CI, −189.75 to 105.37; *p* = 0.899; proportion mediated, −0.16%; 95% CI, −7.56 to 3.86%; *p* = 0.900) (Figure 2; Supplementary Table 5). Similar findings were obtained in the POD1 landmark analysis (ACME, −3.93 days; 95% CI, −197.42 to 110.68; *p* = 0.898) and after additional adjustment for surgical approach and extent of gastrectomy (ACME, −3.30 days; 95% CI, −147.99 to 69.93; *p* = 0.861). These analyses provided no statistical evidence that POD1 GLR mediated the association between VATI and OS.

**Figure 2:**
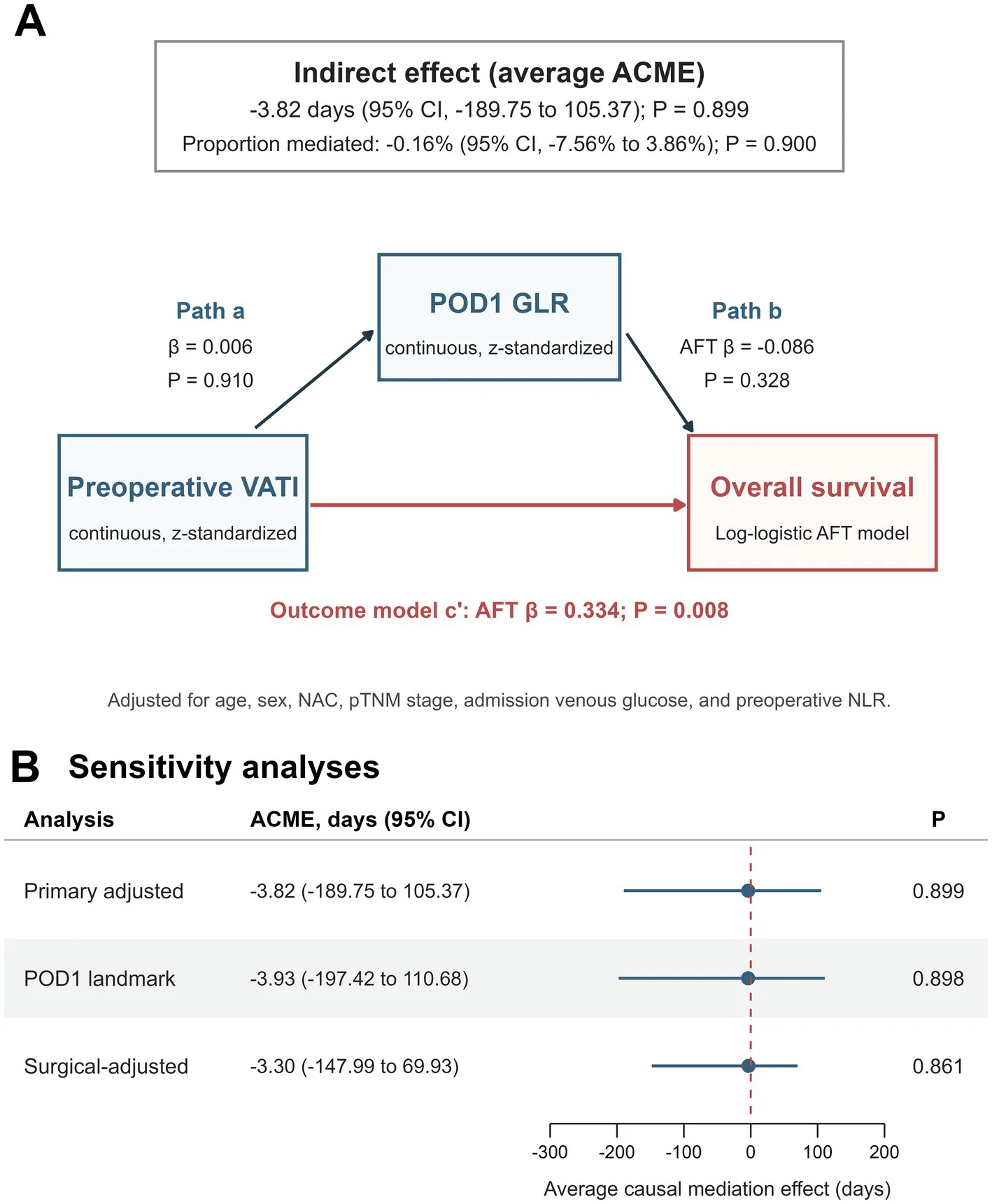
Causal mediation analysis of the association between preoperative VATI, POD1 GLR, and overall survival. **(A)** Primary causal mediation model. Preoperative VATI and POD1 GLR were analyzed as standardized continuous variables. Path a was estimated using a linear regression model, whereas paths b and c’ were estimated using a log-logistic accelerated failure-time (AFT) survival model. Models were adjusted for age, sex, neoadjuvant chemotherapy (NAC), pathological TNM stage, admission venous blood glucose, and preoperative NLR. **(B)** Sensitivity analyses using a POD1 landmark definition and additional adjustment for surgical approach and gastrectomy extent. ACME estimates are expressed on the survival-time scale and should not be interpreted as hazard ratios. AFT, accelerated failure time; ACME, average causal mediation effect; CI, confidence interval; VATI, visceral adipose tissue index; GLR, glucose-to-lymphocyte ratio; NLR, neutrophil-to-lymphocyte ratio; OS, overall survival.

### Construction and validation of the nomogram

A TGV nomogram incorporating pTNM stage, NAC, GLR, and VATI was developed (Figure 3A). The TGV model yielded Harrell’s C-indices of 0.765 in the training cohort and 0.697 in the internal validation cohort. Calibration analysis showed generally acceptable agreement between predicted and observed 3- and 5-year survival probabilities in the training and internal validation cohorts (Figures 3B,C).

**Figure 3:**
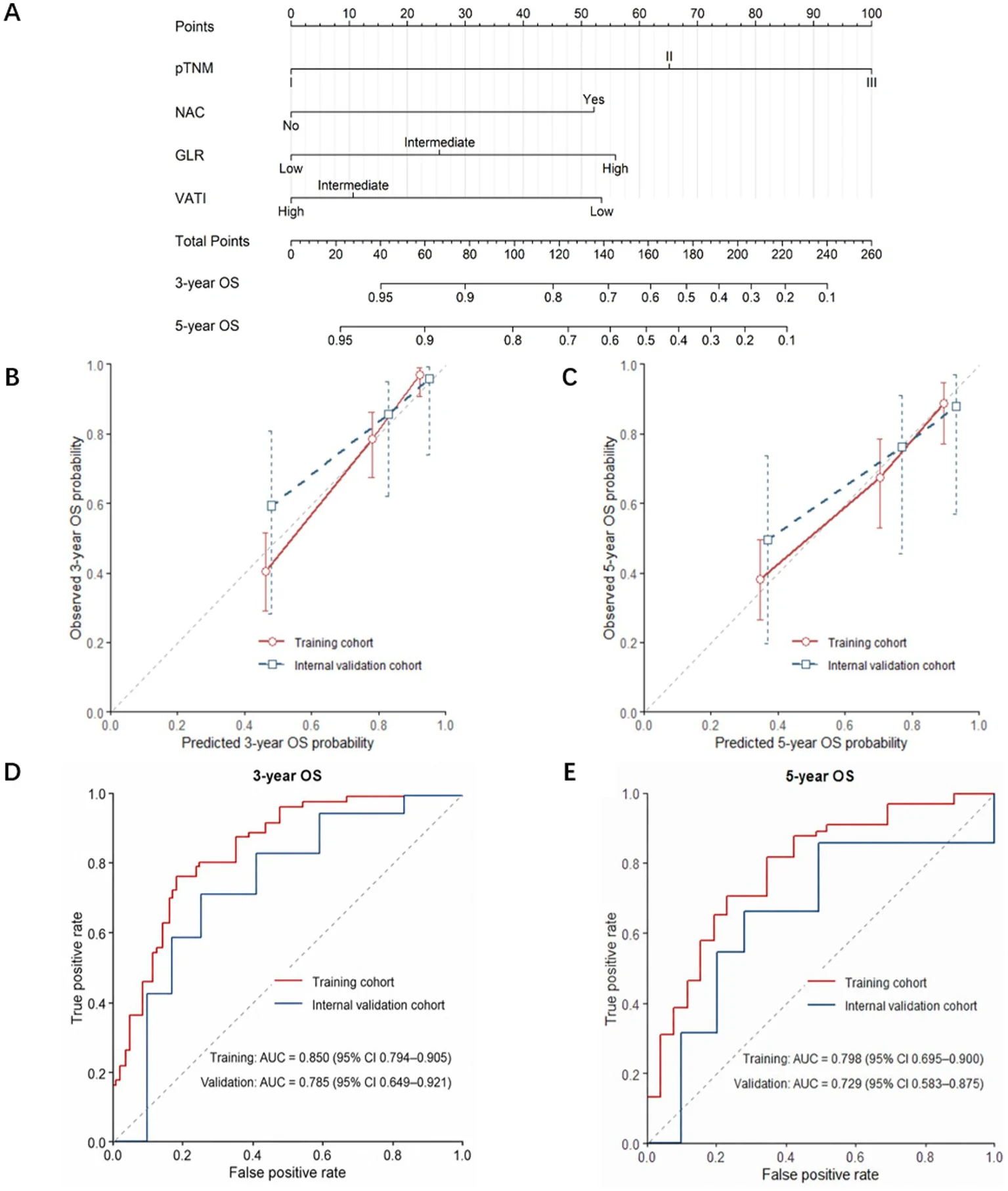
Development and validation of the TGV prognostic nomogram for overall survival prediction. **(A)** TGV nomogram integrating pTNM stage, neoadjuvant chemotherapy (NAC), POD1 glucose-to-lymphocyte ratio (GLR), and preoperative visceral adipose tissue index (VATI) for individualized prediction of 3- and 5-year overall survival (OS). **(B,C)** Calibration curves evaluating the agreement between predicted and observed probabilities for 3-year **(B)** and 5-year **(C)** OS in the training and internal validation cohorts. **(D,E)** Time-dependent receiver operating characteristic (ROC) curves assessing the discrimination performance of the TGV model for 3-year **(D)** and 5-year **(E)** OS prediction in the training and internal validation cohorts. AUC, area under the curve; GLR, glucose-to-lymphocyte ratio; NAC, neoadjuvant chemotherapy; OS, overall survival; ROC, receiver operating characteristic; TGV, TNM stage–GLR–VATI model; VATI, visceral adipose tissue index.

Time-dependent ROC analysis showed 3- and 5-year AUCs of 0.850 and 0.798, respectively, in the training cohort and 0.785 and 0.729 in the internal validation cohort (Figures 3D,E). The corresponding AUCs for TNM staging alone were 0.769 and 0.702 in the training cohort and 0.782 and 0.642 in the internal validation cohort. In the internal validation cohort, the TGV model showed numerically higher discrimination than TNM alone, TNM + NLR, and TNM + PLR across all three metrics, whereas TNM + PNI yielded marginally higher point estimates for the C-index and 3- and 5-year AUCs (Supplementary Table 6).

Training-cohort DCA suggested a higher net benefit for the TGV model than TNM staging across much of the evaluated threshold range at both 3 and 5 years (Figures 4A,B). Additionally, an online tool was developed^1^ to rapidly predict patient prognosis by incorporating NAC, pTNM, GLR, and VATI (Figure 5).

**Figure 4:**
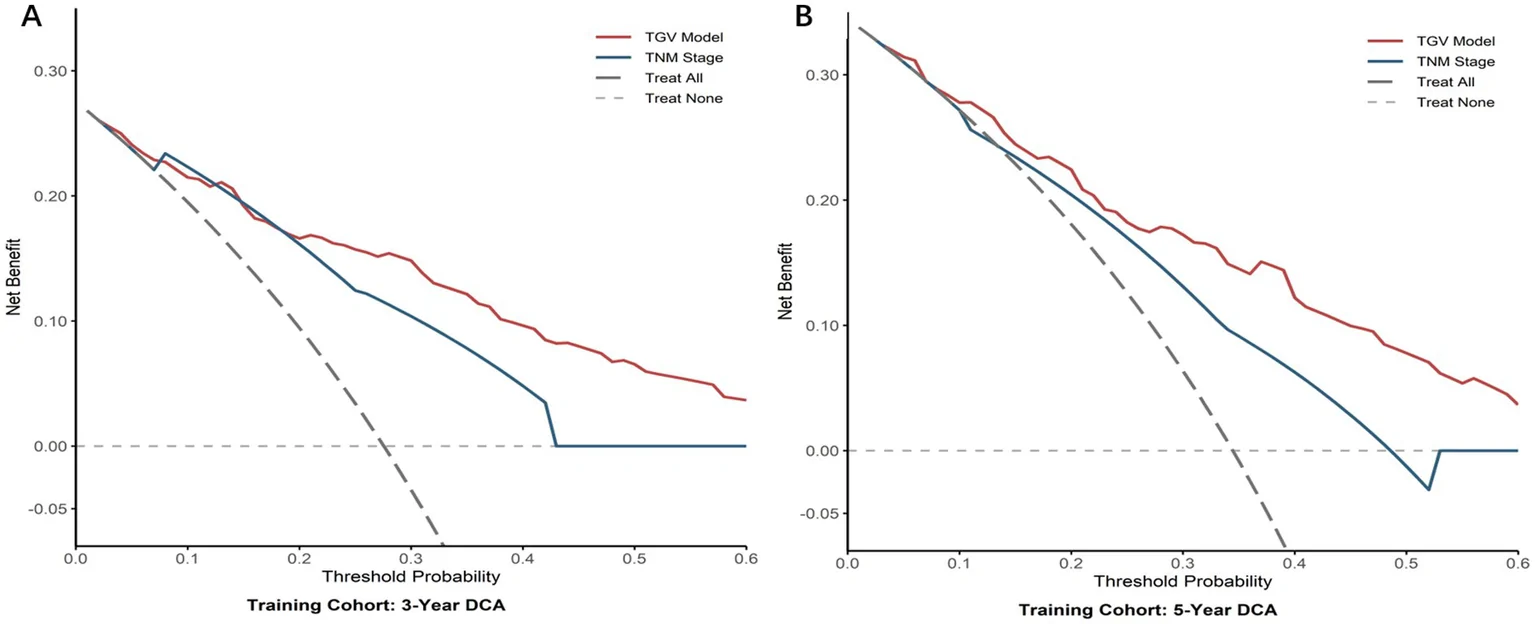
Decision curve analysis of the TGV model and TNM stage for predicting overall survival in gastric cancer patients. **(A)** Three-year decision curve analysis comparing the net benefit of the TGV model, TNM stage, treat-all strategy, and treat-none strategy in the training cohort. **(B)** Five-year decision curve analysis evaluating the clinical utility of the TGV model and TNM stage in the training cohort. DCA, decision curve analysis; OS, overall survival; TGV, TNM stage–GLR–VATI model.

**Figure 5:**
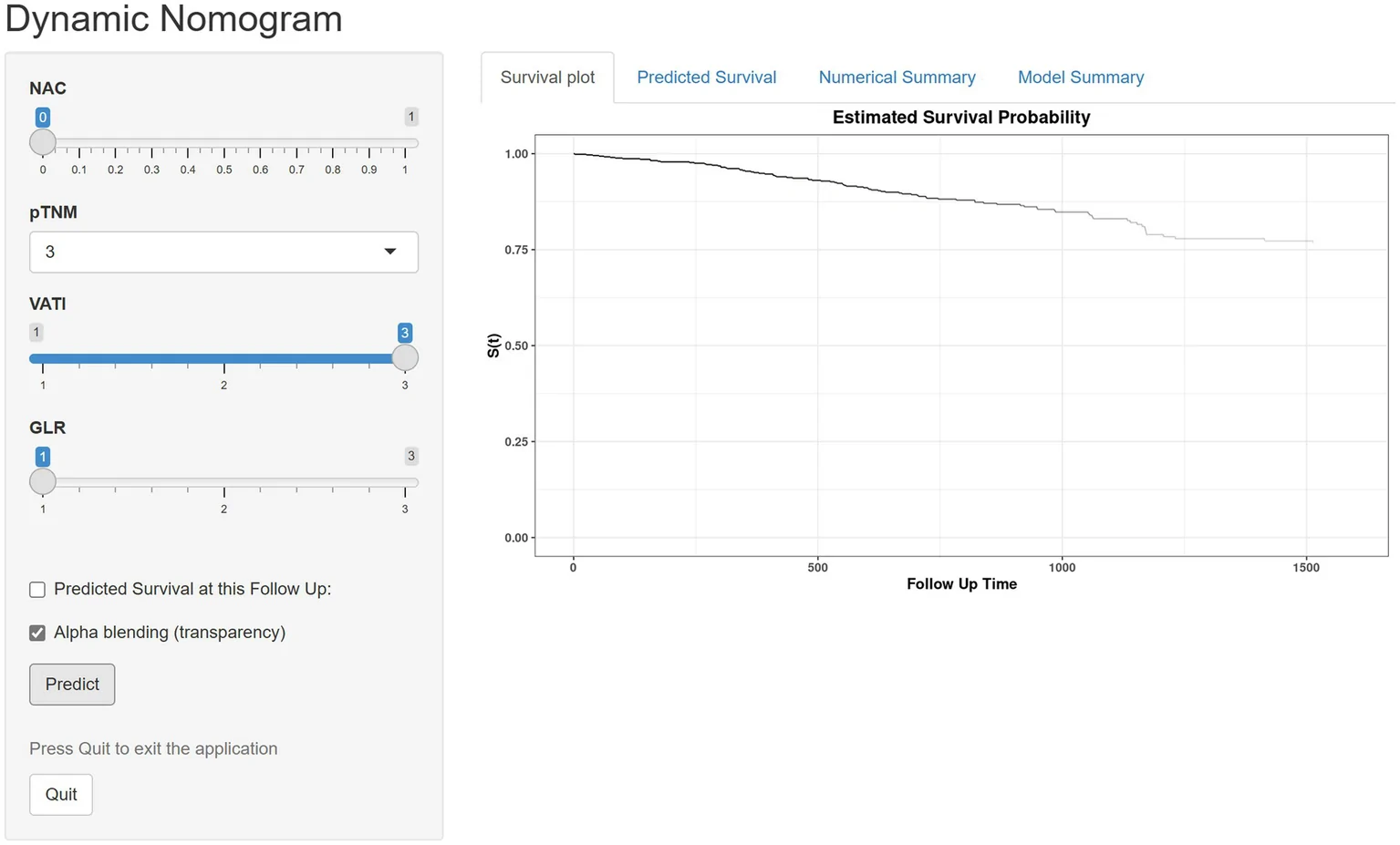
The intuitive interface of the online dynamic TGV nomogram.

## Discussion

This study identified POD1 GLR as a prognostic marker, with higher GLR being significantly associated with poorer OS in patients with GC. The selection of POD1 for measuring GLR is consistent with previous clinical evidence. A comprehensive review reported that 81.5% of studies employing a single postoperative time point selected POD1 (22). Furthermore, POD1 biomarkers have been shown to capture the peak surgical stress response and provide greater prognostic information compared with later time points such as POD3 (23). In a large cohort of 1,635 GC patients, Yamamoto et al. (2025) similarly reported greater prognostic value for POD1 than for POD3 measurements (24). Thus, POD1 may provide a clinically relevant window for assessing the early postoperative stress response. Our findings are also consistent with a recent meta-analysis, which demonstrated that GLR reflects the metabolic, inflammatory, and immune status in patients with solid tumors (15). From a pathophysiological perspective, tumor cells enhance glucose uptake through Warburg effect-mediated metabolic reprogramming. This process not only sustains tumor proliferation but also may contribute to an immunosuppressive microenvironment through nutrient competition and lactate accumulation (25). Peripheral blood lymphocyte count serves as a surrogate marker for host immune function, and lower lymphocyte counts have been associated with poorer prognosis across various malignancies (26). In the specific context of surgical trauma, elevated GLR on POD1 reflects postoperative stress-induced hyperglycemia accompanied by lymphocytopenia, and may therefore capture stress-induced insulin resistance and impaired immune function (18, 27). Acute postoperative neuroendocrine responses, characterized by increased cortisol and catecholamine levels, may also contribute to transient lymphocyte redistribution and apoptosis through glucocorticoid-related pathways (27). Previous studies have shown that severe postoperative hyperglycemia is associated with postoperative complications and has been linked to HIF-1α overexpression and the release of pro-inflammatory cytokines, such as IL-6 and TNF-α (10, 11). Moreover, iatrogenic hyperglycemia, related to excessive parenteral glucose administration has been associated with increased postoperative mortality (10, 14). On the other hand, maintaining lymphocyte levels is associated with improved prognosis through enhanced immune surveillance (26). Therefore, POD1 GLR may serve as an accessible marker of acute postoperative immunometabolic stress after gastrectomy. These findings support further investigation of perioperative glucose metabolism and immune status for postoperative risk assessment.

Recent studies suggest that elevated VATI may be linked to systemic inflammation and metabolic dysfunction (16, 28). Visceral adipose tissue (VAT) is an active endocrine tissue that secretes adipokines and participates in metabolic and immune regulation (29, 30). However, in this study, high VATI was positively associated with prolonged OS in GC patients, consistent with previous studies (16, 31–33), which may be consistent with the “obesity paradox” in cancer epidemiology. The prognostic impact of visceral adiposity may vary according to population characteristics, metabolic phenotype and cancer type (29–31, 34). This may be particularly relevant in relatively lean populations, in whom greater adipose reserves may reflect preserved nutritional status rather than excessive adiposity. In our cohort of non-diabetic patients (mean BMI 22.03 kg/m^2^), the high-VATI group had a mean BMI of 23.81 kg/m^2^ and higher preoperative serum albumin levels than the low-VATI group, supporting an association between greater VATI and better nutritional reserve. Recent studies indicate that the “non-sarcopenic” phenotype, characterized by adequate muscle and fat reserves, is associated with better clinical outcomes in gastrointestinal malignancies (29). In GC, where cancer-associated wasting may progressively deplete energy reserves, greater visceral fat stores may therefore provide an additional indicator of preserved metabolic reserve (30). Consistent with this interpretation, in our relatively lean, non-diabetic cohort, higher VATI was associated with higher BMI, serum albumin, and SMI, but not with preoperative systemic inflammatory markers, suggesting that greater visceral fat stores may reflect preserved nutritional and energy reserves rather than overt metabolic dysfunction. This pattern may be conceptually consistent with a metabolically favorable adiposity phenotype, although MHO could not be formally defined in the present cohort (29, 30). Moreover, VATI was not significantly associated with preoperative NLR, LMR, or PLR. Thus, within this specific population, higher VATI may primarily reflect preserved nutritional and metabolic reserves rather than systemic inflammatory burden (16, 18, 28, 30, 34).

In the cohort, VATI showed a significant positive correlation with SMI, BMI, and albumin levels. Notably, SMI increased across VATI categories, suggesting that elevated visceral adiposity was accompanied by greater skeletal muscle reserve rather than a sarcopenic phenotype. In multivariable analysis, low VATI remained independently associated with poorer OS (HR = 2.341, 95% CI: 1.058–5.178, *p* = 0.036), while SMI did not reach statistical significance. This finding is consistent with cancer cachexia literature, which indicates that visceral fat depletion may precede skeletal muscle loss, suggesting that VATI may be a sensitive marker of early metabolic depletion in malignancy (35). This concept is also consistent with multidimensional cachexia-related indices, such as the Cancer Cachexia Index, which integrates skeletal muscle mass, serum albumin, and systemic inflammation for risk assessment (36). Notably, the data collection period coincided with the COVID-19 pandemic, which disrupted healthcare services globally. Previous studies have suggested that pandemic-related diagnostic delays contributed to later disease stages and worsened nutritional status in GC patients (37). However, supplementary analysis showed no significant differences in BMI, VATI, PNI, or pTNM stage distribution between the pre-pandemic and pandemic periods, suggesting that the baseline nutritional and clinicopathological characteristics of the surgical cohort remained broadly comparable across these periods.

Mediation analysis showed no statistically significant indirect effect of POD1 GLR on the association between VATI and OS (ACME, −3.82 days; 95% CI, −189.75 to 105.37; *p* = 0.899). This contrasts with our previous study, in which postoperative blood glucose showed a statistically significant indirect effect on the VATI–OS association (−12.9%, *p* = 0.024) (18). This discrepancy may reflect differences in the biological information captured by postoperative blood glucose and GLR, the latter integrating both glycemic and immune status. Importantly, the absence of a statistically detectable indirect effect should not be interpreted as evidence that VATI and GLR operate through biologically independent mechanisms. Rather, no statistical evidence supporting a GLR-mediated pathway between VATI and OS was identified in this cohort. Thus, compared with our previous glucose-based analysis, the present study reassessed the VATI–survival mediation hypothesis using POD1 GLR and extended the analysis by developing and internally validating a prognostic model integrating GLR and VATI with established clinicopathological factors. These findings represent an extension of the previous analysis rather than evidence derived from an independent patient cohort.

Given that TNM staging primarily reflects anatomical tumor burden, it does not fully capture host metabolic and nutritional heterogeneity (3). The TGV model integrated pTNM stage, NAC, GLR, and VATI to capture both tumor-related and host-related prognostic information. Although the TGV model showed numerically higher discrimination than TNM alone, TNM + NLR, and TNM + PLR across all three metrics in the internal validation cohort, TNM + PNI yielded marginally higher point estimates. Given the limited validation sample and wide, overlapping confidence intervals, these findings should be interpreted cautiously, and the incremental prognostic and clinical value of TGV requires confirmation in independent external cohorts. In support of the importance of metabolic factors in prognosis, Wang et al. demonstrated the contribution of metabolic indicators to outcome prediction for non-diabetic patients using SHAP analysis (38). To improve clinical applicability, we developed an online prognostic tool to facilitate individualized risk estimation. In this cohort, NAC was associated with poorer long-term survival. However, this association should not be interpreted as evidence of a detrimental effect of NAC, because treatment allocation was not randomized and may have been influenced by confounding by indication. Patients receiving NAC had higher rates of total gastrectomy (65.5% vs. 35.4%, *p* < 0.001) and open surgical approach (75.9% vs. 60.1%, *p* = 0.004), suggesting differences in disease complexity and treatment selection between the groups. Therefore, the observed association between NAC and OS may partly reflect the underlying disease characteristics of patients selected for neoadjuvant treatment rather than a causal effect of NAC itself. Future studies incorporating pretreatment clinical stage, NAC regimen, treatment cycles, and pathological response are needed to clarify this association (39–41).

This study has several limitations. First, as a single-center retrospective study with a small sample size, it may be subject to bias. Second, the COVID-19 triage protocols likely excluded severely malnourished or advanced-stage patients, and the cohort comprised non-diabetic GC patients, limiting the generalizability of the findings. In addition, incomplete availability of admission HbA1c measurements means that previously undiagnosed diabetes cannot be completely excluded. Third, GLR was measured on POD1, and may be influenced by surgical stress; Prospective studies are needed to assess the stability of its dynamic changes over time. The precise number of chemotherapy cycles administered remains undetermined. Future prospective cohorts are required to assess the influence of NAC regimens and cycle durations on patient outcomes. Although the TGV model showed potential for individualized risk stratification, its clinical utility and generalizability remain to be established through external validation. Finally, further research is needed to explore the molecular mechanisms through which VATI influences GC progression and prognosis.

## Conclusion

In conclusion, low preoperative VATI and high POD1 GLR were independently associated with poorer OS in non-diabetic GC patients. Mediation analysis provided no statistically significant evidence that POD1 GLR mediated the association between VATI and OS. A prognostic model integrating pTNM stage, NAC, GLR, and VATI was developed and internally validated, but its incremental prognostic value over established TNM-based models requires confirmation in independent external cohorts. These findings support further evaluation of perioperative metabolic, immune, and body-composition assessment for risk stratification in GC.

## Data Availability

The raw data supporting the conclusions of this article will be made available by the authors, without undue reservation.

## Footnotes

1
https://laimin12.shinyapps.io/DynNomapp/
