# Supplementary material for: Prognostic value of postoperative glucose-to-lymphocyte ratio and visceral fat index in non-diabetic gastric cancer patients: a mediation and modeling study
Source: Front Nutr. 2026 Oct 23;13:1838308. doi: 10.3389/fnut.2026.1838308 (PMC13739479; doi:10.3389/fnut.2026.1838308)
Supplement: Supplementary file 1 [file Table_1.docx]

**Supplement Table 1**. Comparison of surgical characteristics between the NAC and non-NAC groups

| **Variable** | **NAC**  **（n=116）** | **No-NAC**  **（n=223）** | **P** |
| --- | --- | --- | --- |
| Operation approach[n(%)] |  |  | **0.004** |
| Open | 88（75.9） | 134（60.1） |  |
| Laparoscopic | 28（24.1） | 89（39.9） |  |
| Reconstruction[n(%)] |  |  | **0.005** |
| Billroth Ⅰ | 2 (1.7) | 16 (7.2) |  |
| Billroth Ⅱ | 14 (12.1) | 49 (22.0) |  |
| Roux-en-Y | 100 (86.2) | 158 (70.8) |  |
| Gastrectomy scope [n(%)] |  |  | **0.000** |
| Proximal | 0 (0) | 4 (1.8) |  |
| Distal | 40 (34.5) | 140 (62.8) |  |
| Total | 76 (65.5) | 79 (35.4) |  |

NAC, Neoadjuvant chemotherapy; No-NAC, No Neoadjuvant chemotherapy.

**Supplementary Table 2.** Comparison of baseline nutritional and clinicopathological characteristics between the pre-pandemic and pandemic cohorts

| **Variable** | **Pre-pandemic (n=181)** | **Pandemic (n=158)** | **P** |
| --- | --- | --- | --- |
| BMI [n (%)] |  |  | **0.172** |
| <18.5 | 24 (13.3%) | 14 (8.9%) |  |
| 18.5–24 | 118 (65.2%) | 98 (62.0%) |  |
| ≥24 | 39 (21.5%) | 46 (29.1%) |  |
| VATI [n (%)] |  |  | **0.081** |
| Low | 45 (24.9%) | 24 (15.2%) |  |
| Intermediate | 61 (33.7%) | 57 (36.1%) |  |
| High | 75 (41.4%) | 77 (48.7%) |  |
| PNI [n (%)] |  |  | **0.561** |
| Low | 33 (18.2%) | 22 (13.9%) |  |
| Intermediate | 93 (51.4%) | 85 (53.8%) |  |
| High | 55 (30.4%) | 51 (32.3%) |  |
| pTNM [n (%)] |  |  | **0.089** |
| I | 62 (34.3%) | 41 (25.9%) |  |
| II | 40 (22.1%) | 50 (31.6%) |  |
| III | 79 (43.6%) | 67 (42.4%) |  |

VATI, visceral fat area index; BMI, body mass index; PNI, prognostic nutritional index; TNM, tumor-node-metastasis.

**Supplementary Table 3.** Comparison of clinicopathological and nutritional characteristics among patients with different VATI levels

| **Variable** | **L-VATI（n=69）** | **M-VATI（n=118）** | **H-VATI（n=152）** | **P** |
| --- | --- | --- | --- | --- |
| BMI[n(%)] |  |  |  | **< 0.001** |
| <18.5 | 19 (27.5%) | 19 (16.1%) | 0 (0.0%) |  |
| 18.5-24 | 49 (71.0%) | 87 (73.7%) | 80 (52.6%) |  |
| ≥24 | 1 (1.4%) | 12 (10.2%) | 72 (47.4%) |  |
| pTNM[n(%)] |  |  |  | 0.467 |
| Ⅰ | 18 (26.1%) | 38 (32.2%) | 47 (30.9%) |  |
| Ⅱ | 16 (23.2%) | 36 (30.5%) | 38 (25.0%) |  |
| Ⅲ | 35 (50.7%) | 44 (37.3%) | 67 (44.1%) |  |
| BMI[M(IQR)] | 19.47（2.46） | 21.16（3.13） | 23.81（3.28） | **< 0.001** |
| SMI(x± s) | 42.74±7.05 | 44.74±7.18 | 47.61±8.47 | **< 0.001** |
| Albumin[M(IQR)] | 42.2（5.56） | 43.0（4.45） | 44.2（4.88） | **< 0.001** |

VATI, visceral fat area index; BMI, body mass index; TNM, tumor-node-metastasis; SMI, Skeletal muscle index.

**Supplementary Table 4.** Comparison of candidate parametric accelerated failure-time distributions for the survival outcome model in the mediation analysis

| **Distribution** | **df** | **Log-likelihood** | **AIC** | **ΔAIC** | **BIC** | **ΔBIC** |
| --- | --- | --- | --- | --- | --- | --- |
| Weibull | 11 | −806.2 | 1634.468 | 4.484 | 1676.554 | 5.059 |
| Log-normal | 11 | −808.0 | 1638.064 | 8.079 | 1680.150 | 8.655 |
| **Log-logistic** | 11 | **−804.0** | **1629.985** | **0.000** | 1672.071 | 0.575 |
| Exponential | 10 | −806.6 | 1633.236 | 3.251 | **1671.496** | **0.000** |

AFT, accelerated failure time; AIC, Akaike information criterion; BIC, Bayesian information criterion; VATI, visceral adipose tissue index; GLR, glucose-to-lymphocyte ratio.

**Supplementary Table 5**. Causal mediation effects in the primary and sensitivity analyses

| **Analysis** | **ACME, days (95% CI)** | **P** | **ADE, days (95% CI)** | **P** | **Total effect, days (95% CI)** | **P** | **Proportion mediated, % (95% CI)** | **P** |
| --- | --- | --- | --- | --- | --- | --- | --- | --- |
| Primary analysis | −3.82 (−189.75 - 105.37) | 0.899 | 2372.5 (562.43 - 11800) | 0.007 | 2368.7 (559.13 - 11804) | 0.008 | −0.16 (−7.56 - 3.86) | 0.900 |
| POD1 landmark analysis | −3.93 (−197.42 - 110.68) | 0.898 | 2442.8 (576.69 - 12742) | 0.007 | 2438.9 (570.69 - 12682) | 0.008 | −0.16 (−7.55 - 3.85) | 0.899 |
| Surgical-adjusted analysis | −3.30 (−147.99 - 69.93) | 0.861 | 2216.4 (531.20 - 10812) | 0.008 | 2213.1 (528.34 - 10972) | 0.008 | −0.15 (−6.05 - 2.99) | 0.860 |

ACME, average causal mediation effect; ADE, average direct effect; CI, confidence interval. VATI and POD1 GLR were standardized before analysis. The POD1 landmark analysis redefined survival time from POD1 onward, whereas the surgically adjusted analysis additionally included surgical approach and extent of gastrectomy. ACME, ADE, and total-effect estimates are expressed on the model-predicted survival-time response scale and should not be interpreted as hazard ratios.

**Supplementary Table 6.** Discriminative performance of the TGV and reference prognostic models in the training and internal validation cohorts

| **Model** | **Training C-index (95% CI)** | **Validation C-index (95% CI)** | **Training 3-y AUC (95% CI)** | **Training 5-y AUC (95% CI)** | **Validation 3-y AUC (95% CI)** | **Validation 5-y AUC (95% CI)** |
| --- | --- | --- | --- | --- | --- | --- |
| TGV | 0.765 (0.716–0.815) | 0.697 (0.562–0.832) | **0.850 (0.794–0.905)** | **0.798 (0.695–0.900)** | **0.785 (0.649–0.921)** | 0.729 (0.583–0.875) |
| TNM | 0.678 (0.629–0.727) | 0.694 (0.557–0.830) | 0.769 (0.707–0.830) | 0.702 (0.583–0.821) | 0.782 (0.647–0.917) | 0.642 (0.400–0.884) |
| TNM+PNI | 0.721 (0.670–0.772) | 0.728 (0.593–0.864) | 0.795 (0.732–0.858) | 0.787 (0.677–0.897) | 0.789 (0.653–0.925) | 0.738 (0.592–0.884) |
| TNM+NLR | 0.698 (0.646–0.750) | 0.688 (0.562–0.813) | 0.775 (0.708–0.842) | 0.712 (0.587–0.836) | 0.762 (0.600–0.924) | 0.604 (0.423–0.785) |
| TNM+PLR | 0.718 (0.664–0.772) | 0.685 (0.562–0.807) | 0.804 (0.741–0.868) | 0.748 (0.639–0.858) | 0.730 (0.583–0.878) | 0.659 (0.512–0.807) |

AUC, area under the curve; CI, confidence interval; C-index, Harrell’s concordance index; GLR, glucose-to-lymphocyte ratio; NAC, neoadjuvant chemotherapy; NLR, neutrophil-to-lymphocyte ratio; PLR, platelet-to-lymphocyte ratio; PNI, prognostic nutritional index; pTNM, pathological tumor–node–metastasis stage; ROC, receiver operating characteristic; TGV, model incorporating pTNM stage, NAC, GLR, and VATI; VATI, visceral adipose tissue index.
